# cocor: A Comprehensive Solution for the Statistical Comparison of Correlations

**DOI:** 10.1371/journal.pone.0121945

**Published:** 2015-04-02

**Authors:** Birk Diedenhofen, Jochen Musch

**Affiliations:** 1 Department of Experimental Psychology, University of Duesseldorf, Duesseldorf, Germany; University of New South Wales, AUSTRALIA

## Abstract

A valid comparison of the magnitude of two correlations requires researchers to directly contrast the correlations using an appropriate statistical test. In many popular statistics packages, however, tests for the significance of the difference between correlations are missing. To close this gap, we introduce cocor, a free software package for the R programming language. The cocor package covers a broad range of tests including the comparisons of independent and dependent correlations with either overlapping or nonoverlapping variables. The package also includes an implementation of Zou’s confidence interval for all of these comparisons. The platform independent cocor package enhances the R statistical computing environment and is available for scripting. Two different graphical user interfaces—a plugin for RKWard and a web interface—make cocor a convenient and user-friendly tool.

## Introduction

Determining the relationship between two variables is at the heart of many research endeavours. In the social sciences, the most popular statistical method to quantify the magnitude of an association between two numeric variables is the Pearson product-moment correlation. It indicates the strength of a linear relationship between two variables, which may be either positive, negative, or zero. In many research contexts, it is necessary to compare the magnitude of two such correlations, for example, if a researcher wants to know whether an association changed after a treatment, or whether it differs between two groups of interest. When comparing correlations, a test of significance is necessary to control for the possibility of an observed difference occurring simply by chance. However, many introductory statistics textbooks [1–5] do not even mention significance tests for correlations. Also in research practice, the necessity of conducting a proper statistical test when comparing the magnitude of correlations is often ignored. For example, in neuroscientific investigations, correlations between behavioral measures and brain areas are often determined to identify the brain area that is most strongly involved in a given task. Rousselet and Pernet [6] criticized that such studies rarely provide quantitative tests of the difference between correlations. Instead, many authors fall prey to a statistical fallacy, and wrongly consider the existence of a significant and a nonsignificant correlation as providing sufficient evidence for a significant difference between these two correlations. Nieuwenhuis, Forstmann, and Wagenmakers [7] also found that, when making a comparison between correlations, researchers frequently interpreted a significant correlation in one condition and a nonsignificant correlation in another condition as providing evidence for different correlations in the two conditions. Such an interpretation, however, is fallacious. As pointed out by Rosnow and Rosenthal [8], “God loves the .06 nearly as much as the .05”. To make a valid, meaningful, and interpretable comparison between two correlations, it is necessary to directly contrast the two correlations under investigation using an appropriate statistical test [7].

Even when recognizing the importance of a formal statistical test of the difference between correlations, the researcher has many different significance tests to choose from, and the choice of the correct method is vital. Before picking a test, researchers have to distinguish between the following three cases: (1) The correlations were measured in two independent groups A and B. This case applies, for example, if a researcher wants to compare the correlations between anxiety and extraversion in two different groups A and B (*ρ*_*A*_ = *ρ*_*B*_). If the two groups are dependent, the relationship between them needs further differentiation: (2) The two correlations can be overlapping (*ρ*_*A*12_ = *ρ*_*A*23_), i.e., the correlations have one variable in common. *ρ*_*A*12_ and *ρ*_*A*23_ refer to the population correlations in group A between variables 1 and 2 and variables 2 and 3, respectively. For instance, a researcher may be interested in determining whether the correlation between anxiety and extraversion is smaller than between anxiety and diligence within the same group A. (3) In the case of two dependent correlations, the two correlations can also be nonoverlapping (*ρ*_*A*12_ = *ρ*_*A*34_), i.e., they have no variable in common. This case applies, for example, if a researcher wants to determine whether the correlation between anxiety and extraversion is higher than the correlation between intelligence and creativity within the same group. A researcher also faces nonoverlapping dependent correlations when investigating whether the correlation between two variables is higher before rather than after a treatment provided to the same group.

For each of these three cases, various tests have been proposed. An overview of the tests for comparing independent correlations is provided in Table 1, and for comparing dependent correlations—overlapping and nonoverlapping—in Tables 2 and 3, respectively. May and Hittner [9] compared the statistical power and Type I error rate of several tests for dependent overlapping correlations, and found no test to be uniformly preferable. Instead, they concluded that the best choice is influenced by sample size, predictor intercorrelation, effect size, and predictor-criterion correlation. Because no clear recommendation for any of these tests can be formulated that applies under all circumstances, and because different methods may be optimal for a research question at hand, it is important that researchers are provided with a tool that allows them to choose freely between all available options. Detailed discussions of the competing tests for comparing dependent overlapping correlations are given in Dunn and Clark [10], Hittner, May, and Silver [11], May and Hittner [9], Neill and Dunn [12], and Steiger [13]. For the case of dependent nonoverlapping correlations, the pros and cons of various tests are discussed in Raghunathan, Rosenthal, and Rubin [14], Silver, Hittner, and May [15], and Steiger [13]. In contrast to most other approaches, Zou [16] has advocated a test that is based on the computation of confidence intervals, which are often regarded as superior to significance testing because they separately indicate the magnitude and the precision of an estimated effect [17, 18]. Confidence intervals can be used to test whether a correlation significantly differs from zero or from some constant, and whether the difference between two correlations exceeds a predefined threshold. Zou’s confidence interval [16] is available for comparisons of independent and dependent correlations with either overlapping or nonoverlapping variables. The tests proposed by Zou [16] have been compared to other confidence interval procedures by Wilcox [19].

**Table 1:** Software implementing tests for comparing two correlations based on independent groups.

**Test**	**psych**	**multilevel**	**Weaver & Wuensch**	**cocor**
Fisher’s [20] *z*	•	•	•	•
Zou’s [16] confidence interval			•	•

**Table 2:** Software implementing tests for comparing two correlations based on dependent groups with overlapping variables.

**Test**	**psych**	**multilevel**	**DEPCORR**	**DEPCOR**	**Weaver & Wuensch**	**cocor**
Pearson and Filon’s [21] *z*						•
Hotelling’s [22] *t*			•			•
Williams’ [23] *t*	•	•	•	•	•	•
Olkin’s [24] *z*			•			•
Dunn and Clark’s [25] *z*			•	•		•
Hendrickson et al.’s [26] modification of Williams’ [23] *t*			•			•
Steiger’s [13] modification of Dunn and Clark’s [25] *z*			•	•		•
Meng, Rosenthal, and Rubin’s [27] *z*			•	•		•
Hittner et al.’s [11] modification of Dunn and Clark’s [25] *z*				•		•
Zou’s [16] confidence interval					•	•

**Table 3:** Software implementing tests for comparing two correlations based on dependent groups with nonoverlapping variables.

**Test**	**psych**	**DEPCOR**	**Weaver & Wuensch**	**cocor**
Pearson and Filon’s [21] *z*			•	•
Dunn and Clark’s [25] *z*	•	•		•
Steiger’s [13] modification of Dunn and Clark’s [25] *z*	•	•		•
Raghunathan, Rosenthal, and Rubin’s [14] modification of Pearson and Filon’s [21] *z*			•	•
Silver, Hittner, and May’s [15] modification of Dunn and Clark’s [25] *z*		•		•
Zou’s [16] confidence interval			•	•

### Existing Software

Many popular statistics programs do not provide any, or only a subset of the significance tests described above. Moreover, existing programs that allow for statistical comparisons between correlations are isolated stand-alone applications and do not come with a graphical user interface (GUI). For example, DEPCOR [28] is a program that is limited to comparisons of two dependent correlations—either overlapping or nonoverlapping. The program is written in Fortran and runs in a DOS command prompt console under the Windows platform. Another available package, DEPCORR [29], is an SAS macro [30] for comparing two dependent overlapping correlations. The latest release of SAS/STAT software (version 9.4) runs on Windows and Linux systems. However, DEPCORR has no GUI and covers only one of the three cases described above. The two packages psych [31] and multilevel [32] for the R programming language [33] also offer functions to compare two dependent or independent correlations. However, each of these functions covers only one or two of the many different available tests of comparison, and there is no GUI available to access the functions of the packages. Weaver and Wuensch [34] recently published thoroughly documented scripts for comparing dependent or independent correlations in SPSS and SAS.

## cocor

**Fig 1:**
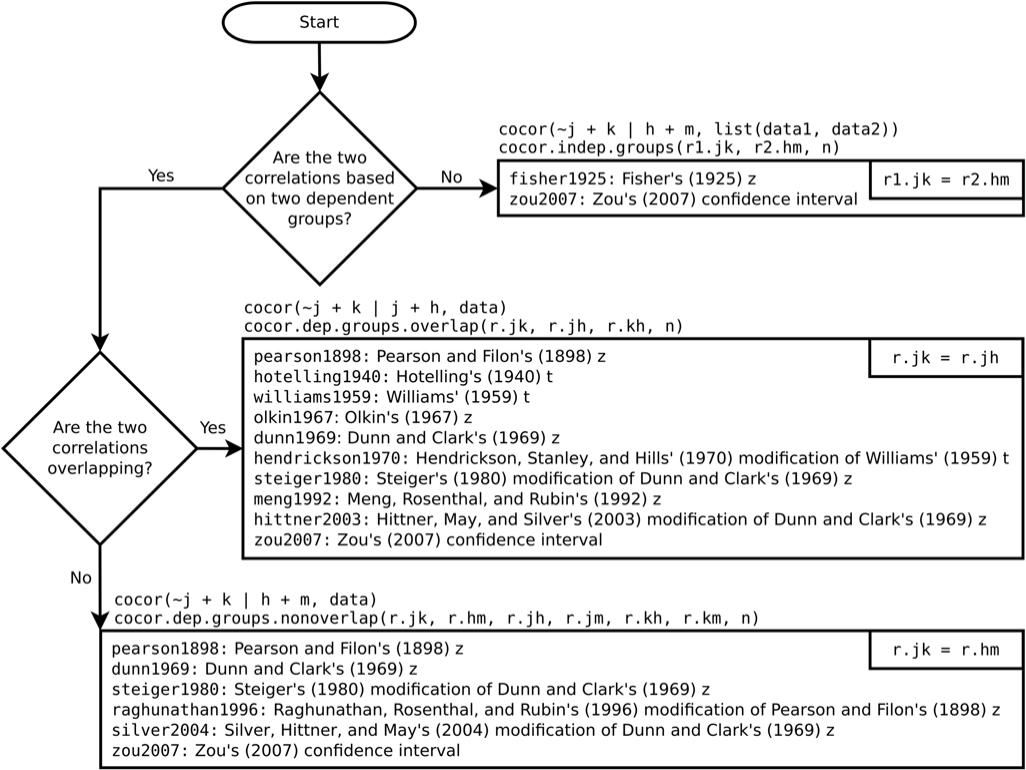
A flowchart of how to use the four main functions of cocor, displaying all available tests. For each case, an example of the formula passed as an argument to the cocor() function and the required correlation coefficients for the functions cocor.indep.groups(), cocor.dep.groups.overlap(), and cocor.dep.groups.nonoverlap() are given. The test label before the colon may be passed as a function argument to calculate specific tests only.

With cocor (version 1.1-0), we provide a comprehensive solution to compare two correlations based on either dependent or independent groups. The cocor package enhances the R programming environment [33], which is freely available for Windows, Mac, and Linux systems and can be downloaded from CRAN (http://cran.r-project.org/package=cocor). All that is needed to install the cocor package is to type install.packages(“cocor”) in the R console, and the functionality of the package is made available by typing library(“cocor”). The function cocor() calculates and compares correlations from raw data. The underlying variables are specified via a formula interface (see Fig. 1). If raw data are not available, cocor offers three functions to compare correlation coefficients that have already been determined. The function cocor.indep.groups() compares two independent correlations, whereas the functions cocor.dep.groups.overlap() and cocor.dep.groups.nonoverlap() compare two dependent overlapping or nonoverlapping correlations, respectively. Internally, cocor() passes the calculated correlations coefficients to one of these three functions. All functions allow to specify the argument null.value to test whether the difference between the correlations exceeds a given threshold using the confidence intervals by Zou [16]. The results are either returned as an S4 object of class cocor whose input and result parameters can be obtained using the get.cocor.input() and get.cocor.results() functions, respectively. Optionally, results may also be returned as a list of class htest. By default, all tests available are calculated. Specific tests can be selected by passing a test label to the function using the test argument. The flowchart in Fig. 1 shows how to access the available tests and lists them with their individual test label (e.g., zou2007). The formulae of all implemented tests are detailed in S1 Appendix.

A comparison of cocor with competing software can be found in Tables 1–3. These tables show that cocor offers a larger variety of tests and a more comprehensive approach than all previous solutions. In particular, cocor is the first R package to implement the tests by Zou [16]. Further unique features of the cocor package are the formula interface for comparing correlations that extracts the correlations from data, and the unified function for statistical tests capable of comparing both, independent and dependent correlations with either overlapping or nonoverlapping variables.

Some limitations of cocor should be acknowledged, however. First, cocor is limited to the comparison of two correlations. The simultaneous comparison of more than two correlations needs tests that go beyond the scope of the present contribution [35–37]. Second, cocor does not allow one to employ structural equation models that are needed for more advanced, but also more complex approaches to the statistical comparison of correlations [38, 39].

### GUIs for cocor

**Fig 2:**
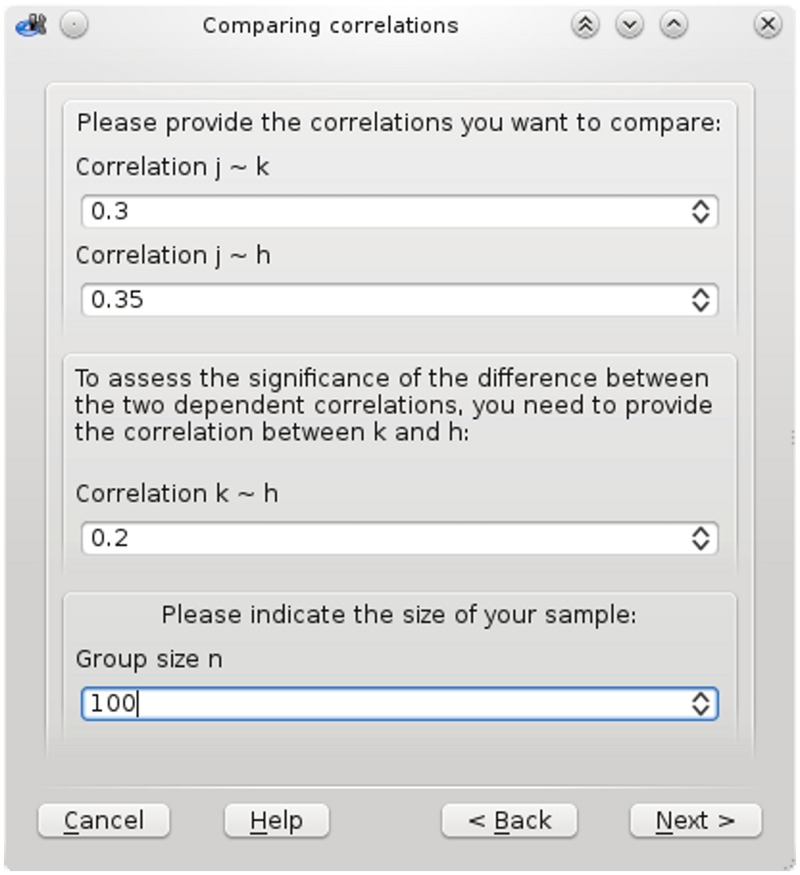
Screenshot of the cocor GUI plugin for RKWard.

**Fig 3:**
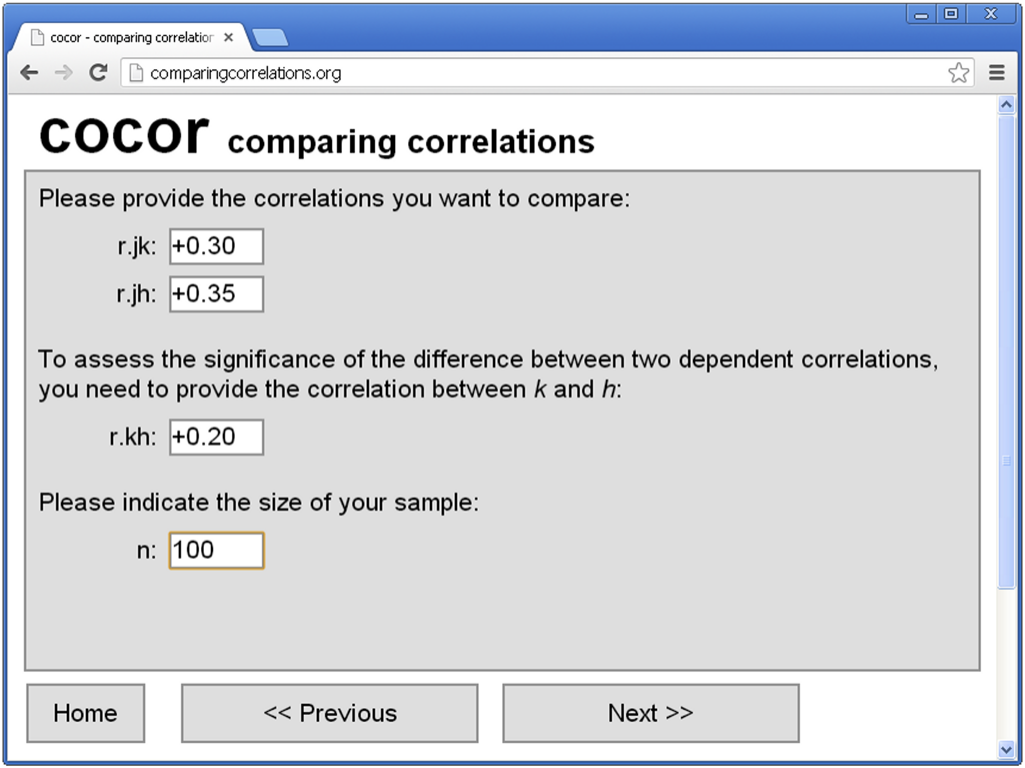
Screenshot of the cocor web interface on http://comparingcorrelations.org.

There are two convenient ways to use cocor via a GUI. First, the package includes a plugin for the platform independent R front-end RKWard [40] (Fig. 2). Second, for those unfamiliar with R, a web interface is also available at http://comparingcorrelations.org (Fig. 3).

Thus, cocor offers the best of two worlds: On the one hand, it has the power of a scripting language with the possibility of automation. On the other hand, the two available GUIs allow even inexperienced users to use cocor in a convenient way. As cocor is embedded in the R environment for statistical computing, it allows for a seamless integration into R analyses. R code can be generated via the GUIs and used for subsequent batch analyses. Since cocor is published under the GNU General Public License (GPL; version 3 or higher), all users are invited to inspect, use, copy, modify, and redistribute the code under the same license.

## Code Examples

In the following, using fictional data, examples are given for all three cases that may occur when comparing correlations.

### Comparison of Two Correlations Based on Independent Groups

The first example presents code for the comparison of the correlations between a score achieved on a logic task (logic) and an intelligence measure A (intelligence.a) in two different groups. Note that the underlying data set (aptitude) is a list that contains two separate data sets.

R> require (“cocor”)

R> data (“aptitude”)

R> cocor (^∼^logic + intelligence.a | logic + intelligence.a, + aptitude)

Results of a comparison of two correlations based on independent groups

Comparison between r1.jk (logic, intelligence.a) = 0.3213 and r2.hm (logic, intelligence.a) = 0.2024

Difference: r1.jk—r2.hm = 0.1189

Data: sample1: j = logic, k = intelligence.a; sample2: h = logic, m = intelligence.a

Group sizes: n1 = 291, n2 = 334

Null hypothesis: r1.jk is equal to r2.hm

Alternative hypothesis: r1.jk is not equal to r2.hm (two-sided)

Alpha: 0.05

fisher1925: Fisher’s z (1925)

z = 1.5869, p-value = 0.1125

Null hypothesis retained

zou2007: Zou’s (2007) confidence interval

95% confidence interval for r1.jk—r2.hm: -0.0281 0.2637

Null hypothesis retained (Interval includes 0)

In this example, the test result indicates that the difference between the two correlations r1.jk and r2.hm is not significant, and the null hypothesis cannot be rejected. Alternatively, the same comparison can also be conducted based on the correlation coefficients and the group sizes using the function cocor.indep.groups().

R> cocor.indep.groups (r1.jk = 0.3213, r2.hm = 0.2024, n1 = 291, + n2 = 334)

### Comparison of Two Overlapping Correlations Based on Dependent Groups

The second example code determines whether the correlation between a score achieved on general knowledge questions (knowledge) and an intelligence measure A (intelligence.a) differs from the correlation between a score achieved on a logic task (logic) and the same intelligence measure A (intelligence.a) within a group of *n* = 291 persons.

R> cocor (^∼^knowledge + intelligence.a | logic + + intelligence.a, aptitude [[“sample1”]])

Results of a comparison of two overlapping correlations based on dependent groups

Comparison between r.jk (intelligence.a, knowledge) = 0.1038 and r.jh (intelligence.a, logic) = 0.3213

Difference: r.jk—r.jh = -0.2175

Related correlation: r.kh = 0.0257

Data: aptitude [[“sample1”]]: j = intelligence.a, k = knowledge, h = logic

Group size: n = 291

Null hypothesis: r.jk is equal to r.jh

Alternative hypothesis: r.jk is not equal to r.jh (two-sided)

Alpha: 0.05

pearson1898: Pearson and Filon’s z (1898)

z = -2.7914, p-value = 0.0052

Null hypothesis rejected

hotelling1940: Hotelling’s t (1940)

t = -2.8066, df = 288, p-value = 0.0053

Null hypothesis rejected

williams1959: Williams’ t (1959)

t = -2.7743, df = 288, p-value = 0.0059

Null hypothesis rejected

olkin1967: Olkin’s z (1967)

z = -2.7914, p-value = 0.0052

Null hypothesis rejected

dunn1969: Dunn and Clark’s z (1969)

z = -2.7595, p-value = 0.0058

Null hypothesis rejected

hendrickson1970: Hendrickson, Stanley, and Hills’ (1970) modification of Williams’ t (1959)

t = -2.8065, df = 288, p-value = 0.0053

Null hypothesis rejected

steiger1980: Steiger’s (1980) modification of Dunn and Clark’s z (1969) using average correlations

z = -2.7513, p-value = 0.0059

Null hypothesis rejected

meng1992: Meng, Rosenthal, and Rubin’s z (1992)

z = -2.7432, p-value = 0.0061

Null hypothesis rejected

95% confidence interval for r.jk—r.jh: -0.3925 -0.0654

Null hypothesis rejected (Interval does not include 0)

hittner2003: Hittner, May, and Silver’s (2003) modification of Dunn and Clark’s z (1969) using a backtransformed average Fisher’s (1921) Z procedure

z = -2.7505, p-value = 0.0059

Null hypothesis rejected

zou2007: Zou’s (2007) confidence interval

95% confidence interval for r.jk—r.jh: -0.3689 -0.0630

Null hypothesis rejected (Interval does not include 0)

The results of all tests lead to the convergent conclusion that the difference between the two correlations r.jk and r.jh is significant, and the null hypothesis should be rejected. Alternatively, the same comparison can also be conducted based on the correlation coefficients and the group size using the function cocor.dep.groups.overlap().

R> cocor.dep.groups.overlap (r.jk = 0.1038, r.jh = 0.3213, + r.kh = 0.0257, n = 291)

### Comparison of Two Nonoverlapping Correlations Based on Dependent Groups

The third example code tests whether the correlation between a score achieved on general knowledge questions (knowledge) and an intelligence measure A (intelligence.a) differs from the correlation between a score achieved on a logic task (logic) and an intelligence measure B (intelligence.b) within the same group of *n* = 291 persons.

R> cocor (^∼^knowledge + intelligence.a | logic + + intelligence.b, aptitude [[“sample1”]])

Results of a comparison of two nonoverlapping correlations based on dependent groups

Comparison between r.jk (knowledge, intelligence.a) = 0.1038 and r.hm (logic, intelligence.b) = 0.2679

Difference: r.jk—r.hm = -0.164

Related correlations: r.jh = 0.0257, r.jm = 0.1713, r.kh = 0.3213, r.km = 0.4731

Data: aptitude [[“sample1”]]: j = knowledge, k = intelligence.a, h = logic, m = intelligence.b

Group size: n = 291

Null hypothesis: r.jk is equal to r.hm

Alternative hypothesis: r.jk is not equal to r.hm (two-sided)

Alpha: 0.05

pearson1898: Pearson and Filon’s z (1898)

z = -2.0998, p-value = 0.0357

Null hypothesis rejected

dunn1969: Dunn and Clark’s z (1969)

z = -2.0811, p-value = 0.0374

Null hypothesis rejected

steiger1980: Steiger’s (1980) modification of Dunn and Clark’s z (1969) using average correlations

z = -2.0755, p-value = 0.0379

Null hypothesis rejected

raghunathan1996: Raghunathan, Rosenthal, and Rubin’s (1996) modification of Pearson and Filon’s z (1898)

z = -2.0811, p-value = 0.0374

Null hypothesis rejected

silver2004: Silver, Hittner, and May’s (2004) modification of Dunn and Clark’s z (1969) using a backtransformed average Fisher’s (1921) Z procedure

z = -2.0753, p-value = 0.0380

Null hypothesis rejected

zou2007: Zou’s (2007) confidence interval

95% confidence interval for r.jk—r.hm: -0.3162 -0.0095

Null hypothesis rejected (Interval does not include 0)

Also in this example, the test results converge in showing that the difference between the two correlations r.jk and r.hm is significant, and the null hypothesis should be rejected. Alternatively, the same comparison can also be conducted based on the correlation coefficients and the group size using the function cocor.dep.groups.nonoverlap().

R> cocor.dep.groups.nonoverlap (r.jk = 0.1038, r.hm = 0.2679, + r.jh = 0.0257, r.jm = 0.1713, r.kh = 0.3213, + r.km = 0.4731, n = 291)

## Discussion and Summary

In this article, we introduced cocor, a free software package for the R programming language [33]. The cocor package provides a wide range of tests for comparisons of independent and dependent correlations with either overlapping or nonoverlapping variables. Unlike existing solutions, cocor is available for scripting within the R environment, while offering two convenient GUIs: a plugin for RKWard [40] and a web interface. Thus, cocor enables users of all knowledge levels to access a large variety of tests for comparing correlations in a convenient and user-friendly way.

## Supporting Information

S1 Appendix Documentation of All Tests Implemented in cocor. (PDF)

## References

[1] Baguley T. Serious Stats: A Guide to Advanced Statistics for the Behavioral Sciences. Basingstoke, UK: Palgrave Macmillan; 2012.

[2] Bakeman R, Robinson BF. Understanding Statistics in the Behavioral Sciences. Mahwah, NJ: Lawrence Erlbaum; 2005.

[3] Freund JE, Simon GA. Modern Elementary Statistics. 9th ed. London, UK: Prentice-Hall; 1997.

[4] Larson R, Farber B. Elementary Statistics: Picturing the World. 5th ed. Boston, MA: Pearson; 2011.

[5] Wright DB, London K. First (and Second) Steps in Statistics. 2nd ed. London, UK: Sage; 2009.

[6] Rousselet GA, Pernet CR. Improving Standards in Brain-Behavior Correlation Analyses. Front Hum Neurosci. 2012;6: 1–11. 10.3389/fnhum.2012.00119 22563313 PMC3342588

[7] Nieuwenhuis S, Forstmann BU, Wagenmakers EJ. Erroneous Analyses of Interactions in Neuroscience: A Problem of Significance. Nat Neurosci. 2011;14: 1105–1107. 10.1038/nn.2886 21878926

[8] Rosnow RL, Rosenthal R. Statistical Procedures and the Justification of Knowledge in Psychological Science. Am Psychol. 1989;44: 1276–1284. 10.1037/0003-066X.44.10.1276

[9] May K, Hittner JB. Tests for Comparing Dependent Correlations Revisited: A Monte Carlo Study. J Exp Educ. 1997;65: 257–269. 10.1080/00220973.1997.9943458

[10] Dunn OJ, Clark VA. Comparison of Tests of the Equality of Dependent Correlation Coefficients. J Am Stat Assoc. 1971;66: 904–908. 10.1080/01621459.1971.10482369

[11] Hittner JB, May K, Silver NC. A Monte Carlo Evaluation of Tests for Comparing Dependent Correlations. J Gen Psychol. 2003;130: 149–168. 10.1080/00221300309601282 12773018

[12] Neill JJ, Dunn OJ. Equality of Dependent Correlation Coefficients. Biometrics. 1975;31: 531–543. 10.2307/2529435

[13] Steiger JH. Tests for Comparing Elements of a Correlation Matrix. Psychol Bull. 1980;87: 245–251. 10.1037/0033-2909.87.2.245

[14] Raghunathan TE, Rosenthal R, Rubin DB. Comparing Correlated but Nonoverlapping Correlations. Psychol Methods. 1996;1: 178–183. 10.1037/1082-989X.1.2.178

[15] Silver NC, Hittner JB, May K. Testing Dependent Correlations with Nonoverlapping Variables: A Monte Carlo Simulation. J Exp Educ. 2004;73: 53–69. 10.3200/JEXE.71.1.53-70

[16] Zou GY. Toward Using Confidence Intervals to Compare Correlations. Psychol Methods. 2007;12: 399–413. 10.1037/1082-989X.12.4.399 18179351

[17] Cohen J. The Earth Is Round (p *<* .05). Am Psychol. 1994;49: 997–1003. 10.1037/0003-066X.49.12.997

[18] Olkin I, Finn JD. Correlation Redux. Psychol Bull. 1995;118: 155–164. 10.1037/0033-2909.118.1.155

[19] Wilcox RR. Comparing Pearson Correlations: Dealing with Heteroscedasticity and Nonnormality. Commun Stat Simul Comput. 2009;38: 2220–2234. 10.1080/03610910903289151

[20] Fisher RA. Statistical Methods for Research Workers. Edinburgh, Scotland: Oliver and Boyd; 1925 Available: http://psychclassics.yorku.ca. Accessed 21 February 2015.

[21] Pearson K, Filon LNG. Mathematical Contributions to Theory of Evolution: IV. On the Probable Errors of Frequency Constants and on the Influence of Random Selection and Correlation. Philos Trans R Soc Lond A. 1898;191: 229–311.

[22] Hotelling H. The Selection of Variates for Use in Prediction, with Some Comments on the General Problem of Nuisance Parameters. Ann Math Stat. 1940;11: 271–283. 10.1214/aoms/1177731867

[23] Williams EJ. The Comparison of Regression Variables. J R Stat Soc B. 1959;21: 396–399. Available: http://www.jstor.org/stable/2983809. Accessed 21 February 2015.

[24] Olkin I. Correlations Revisited In: Stanley JC, editor. Improving Experimental Design and Statistical Analysis. Chicago, IL: Rand McNally; 1967 pp. 102–128.

[25] Dunn OJ, Clark VA. Correlation Coefficients Measured on the Same Individuals. J Am Stat Assoc. 1969;64: 366–377. 10.1080/01621459.1969.10500981

[26] Hendrickson GF, Stanley JC, Hills JR. Olkin’s New Formula for Significance of r13 vs. r23 Compared with Hotelling’s Method. Am Educ Res J. 1970;7: 189–195. 10.2307/1162159

[27] Meng XL, Rosenthal R, Rubin DB. Comparing Correlated Correlation Coefficients. Psychol Bull. 1992;111: 172–175. 10.1037/0033-2909.111.1.172

[28] Silver NC, Hittner JB, May K. A FORTRAN 77 Program for Comparing Dependent Correlations. Appl Psychol Meas. 2006;30: 152–153. 10.1177/0146621605277132

[29] Hittner JB, May K. DEPCORR: A SAS Program for Comparing Dependent Correlations. Appl Psychol Meas. 1998;22: 93–94. 10.1177/01466216980221010

[30] SI Inc. SAS/STAT Software, Version 9.4. Cary, NC; 2013. Available: http://www.sas.com. Accessed 21 February 2015.

[31] Revelle W. psych: Procedures for psychological, psychometric, and personality research; 2014. R package version 1.4.8. Available: http://cran.R-project.org/package=psych. Accessed 21 February 2015.

[32] Bliese P. multilevel: Multilevel Functions; 2013. R package version 2.5. Available: http://cran.R-project.org/package=multilevel. Accessed 21 February 2015.

[33] R Core Team. R: A Language and Environment for Statistical Computing. Vienna, Austria; 2014. Available: http://www.R-project.org. Accessed 21 February 2015.

[34] Weaver B, Wuensch KL. SPSS and SAS programs for comparing Pearson correlations and OLS regression coefficients. Behav Res Methods. 2013;45: 880–895. 10.3758/s13428-012-0289-7 23344734

[35] Levy KJ. A Multiple Range Procedure for Independent Correlations. Educ Psychol Meas. 1976;36: 27–31. 10.1177/001316447603600103

[36] Paul SR. A Multiple Range Procedure for Independent Correlations. Can J Stat. 1989;17: 217–227. 10.2307/3314850

[37] Silver NC, Zaikina H, Hittner JB, May K. INCOR: A Computer Program for Testing Differences among Independent Correlations. Mol Ecol Resour. 2008;8: 763–764. 10.1111/j.1755-0998.2008.02107.x 21585885

[38] Cheung MWL, Chan W. Testing Dependent Correlations via Structural Equation Modeling. Org Res Methods. 2004;7: 206–223. 10.1177/1094428104264024

[39] Cheung MWL. Constructing Approximate Confidence Intervals for Parameters with Structural Equation Models. Struct Equ Modeling. 2009;16: 267–294. 10.1080/10705510902751291

[40] Rödiger S, Friedrichsmeier T, Kapat P, Michalke M. RKWard: A Comprehensive Graphical User Interface and Integrated Development Environment for Statistical Analysis with R. J Stat Softw. 2012;49: 1–34. Available: http://www.jstatsoft.org/v49/i09. Accessed 21 February 2015.

