# Correction: cocor: A Comprehensive Solution for the Statistical Comparison of Correlations

**DOI:** 10.1371/journal.pone.0131499

**Published:** 2015-06-26

**Authors:** The PLOS ONE Staff

The URL in the Data Availability statement for this paper is incorrect. The correct statement is: “Data Availability Statement: The cocor R package can be downloaded from http://cran.r-project.org/package=cocor. A web front-end to conveniently access the functionality of the cocor package is available at http://comparingcorrelations.org.” The publisher apologizes for the error.

There is an error in the URL in the second sentence of the subsection “cocor” in the Introduction. The correct sentence should be: “The cocor package enhances the R programming environment [33], which is freely available for Windows, Mac, and Linux systems and can be downloaded from CRAN (http://cran.r-project.org/package=cocor).” The publisher apologizes for the error.

There is an error in the URL in reference 31 of the References. The correct reference should be: “Revelle W. psych: Procedures for psychological, psychometric, and personality research; 2014. R package version 1.4.8. Available: http://cran.R-project.org/package=psych. Accessed 21 February 2015.” The publisher apologizes for the error.

There is an error in the URL in reference 32 of the References. The correct reference should be: “Bliese P. multilevel: Multilevel Functions; 2013. R package version 2.5. Available: http://cran.R-project.org/package=multilevel. Accessed 21 February 2015.” The publisher apologizes for the error.

There are several errors in the “Comparison of Two Correlations Based on Independent Groups” subsection of the Code Examples section. The publisher apologizes for the error. Please view the correct code here. Figs 4 and 5

**Fig 4:**
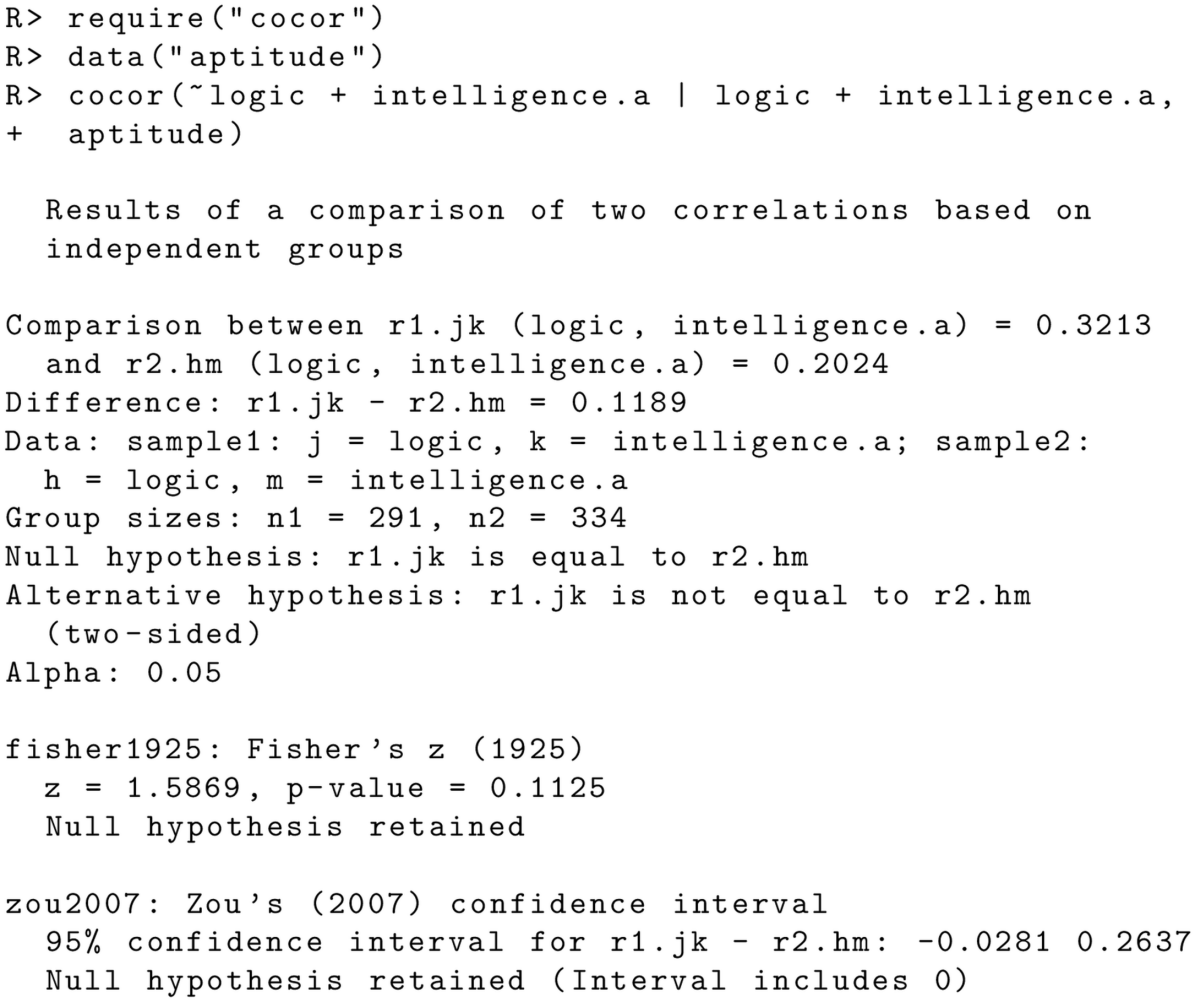


**Fig 5:**
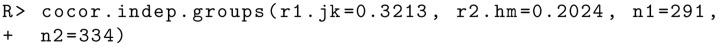


There are several errors in the “Comparison of Two Overlapping Correlation Based on Dependent Groups” subsection of the Code Examples section. The publisher apologizes for the error. Please view the correct code here. Figs 6 and 7

**Fig 6:**
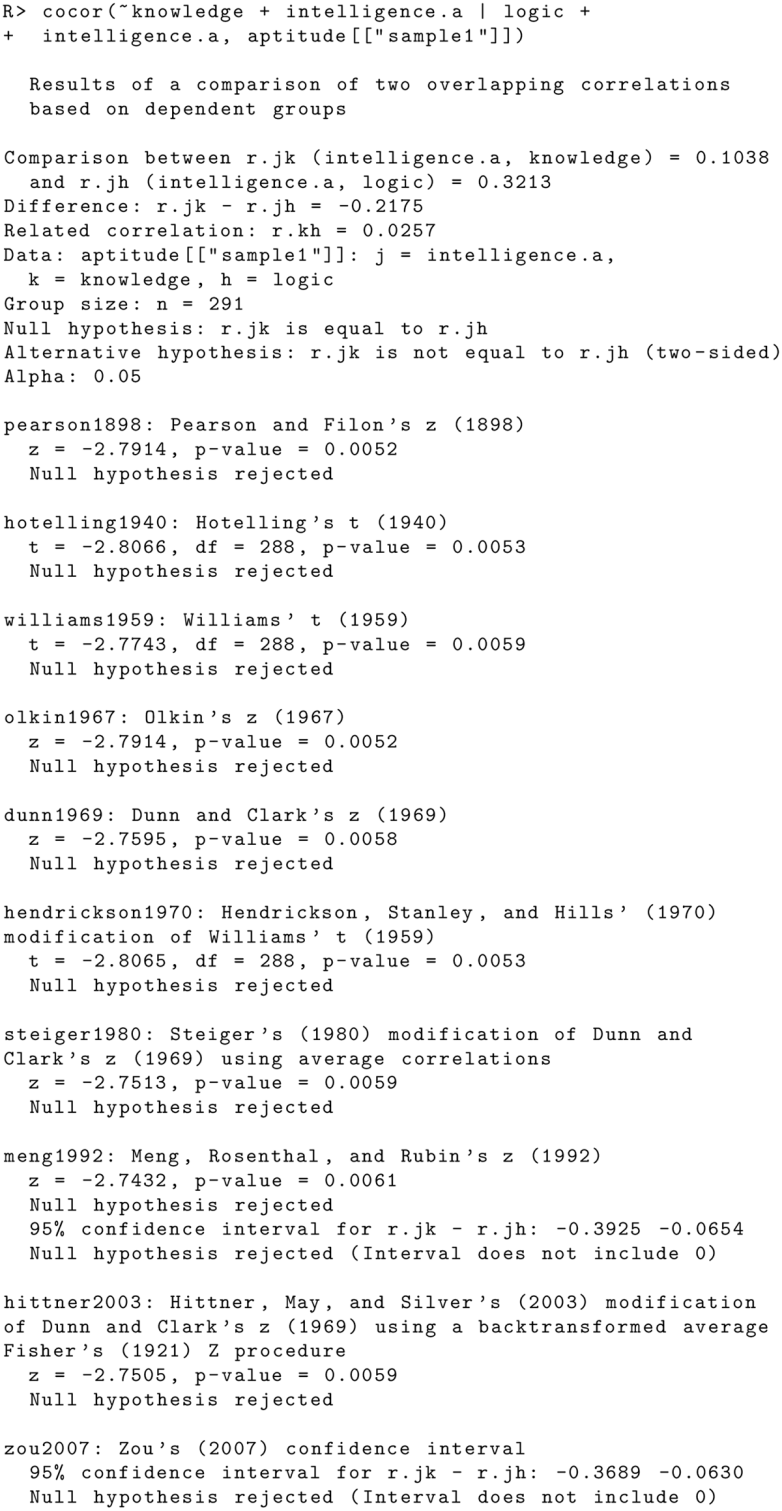


**Fig 7:**
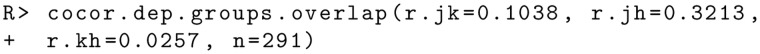


There are several errors in the “Comparison of Two Nonoverlapping Correlations Based on Dependent Groups” subsection of the Code Examples section. The publisher apologizes for the error. Please view the correct code here. Figs 8 and 9

**Fig 8:**
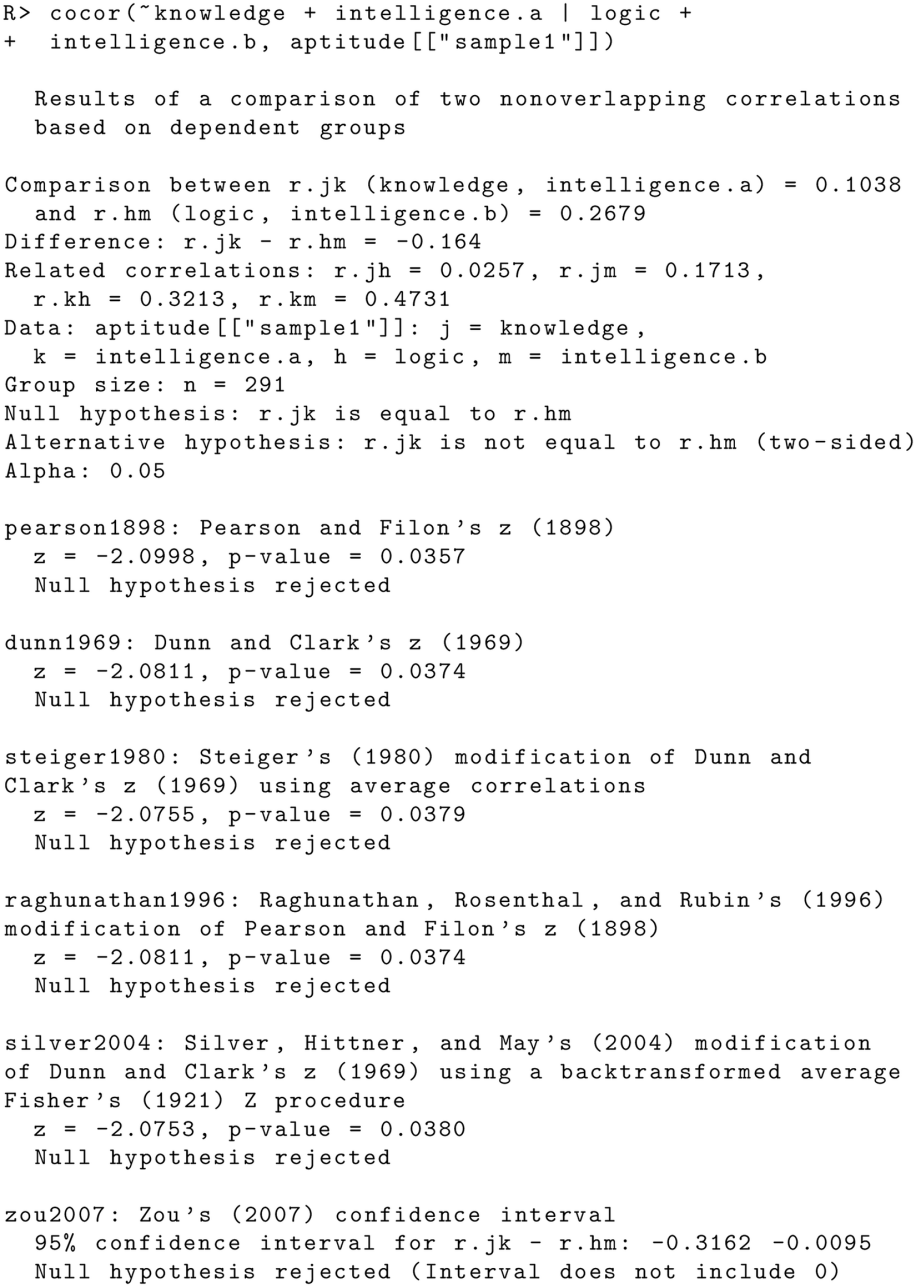


**Fig 9:**
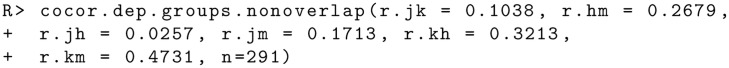


In the Supporting Information file S1 Appendix, there are errors in Equations 4, 32, and 51. These equations should contain a “+” sign before the square root sign instead of a “-” sign. The publisher apologizes for the error. Please view the correct S1 Appendix below.

## Supporting Information

S1 Appendix Documentation of All Tests Implemented in cocor. (PDF)
