# Supplementary material for: Correction: cocor: A Comprehensive Solution for the Statistical Comparison of Correlations
Source: PLoS One. 2015 Jun 26;10(6):e0131499. doi: 10.1371/journal.pone.0131499 (PMC4482741; doi:10.1371/journal.pone.0131499)
Supplement: S1 Appendix — (PDF) [file pone.0131499.s001.pdf]

S1 Appendix. Documentation of All Tests Implemented in cocor

This Appendix is part of the article *cocor: A Comprehensive Solution for the Statistical Comparison of Correlations* by Birk Diedenhofen[1] and Jochen Musch published in PLOS ONE. In the following, the formulae of all tests implemented in the R package [1] `cocor` (version 1.1-0) are provided. z statistics are based on a normal distribution, whereas t statistics rely on a Student's t-distribution with given degrees of freedom. Some tests make use of Fisher's [2, p 26] r-to-Z transformation:

$$Z = \frac{1}{2}(ln(1+r) - ln(1-r)). \tag{1}$$

Tests for Comparison of Two Correlations Based on Independent Groups

The function `cocor.indep.groups()` implements tests for the comparison of two correlations based on independent groups.

fisher1925: Fisher's [3] z

This significance test was first described by Fisher [3, pp 161–168] and its test statistic z is calculated as

$$z = \frac{Z_1 - Z_2}{\sqrt{\frac{1}{n_1-3} + \frac{1}{n_2-3}}}. \tag{2}$$

Z_1 and Z_2 are the two Z transformed correlations that are being compared. n_1 and n_2 specify the size of the two groups the correlations are based on. Equation 2 is also given for example in Peters and van Voorhis [4, p 188] and Cohen, Cohen, West, and Aiken [5, p 49, formula 2.8.11].

zou2007: Zou's [6] confidence interval

This test calculates the confidence interval of the difference between the two correlation coefficients r_1 and r_2. If the confidence interval includes zero, the null hypothesis that the two correlations are equal must be retained. If the confidence interval does not include zero, the null hypothesis has to be rejected. A lower and upper bound for the interval (L and U, respectively) is given by

$$L = r_1 - r_2 - \sqrt{(r_1 - l_1)^2 + (u_2 - r_2)^2} \tag{3}$$

[1]corresponding author, e-mail: birk.diedenhofen@uni-duesseldorf.de

and

$$U = r_1 - r_2 + \sqrt{(u_1 - r_1)^2 + (r_2 - l_2)^2} \tag{4}$$

[6, p 409]. A lower and upper bound for the confidence interval of r_1 (l_1 and u_1) and r_2 (l_2 and u_2) are calculated as

$$l = \frac{exp(2l') - 1}{exp(2l') + 1}, \tag{5}$$

$$u = \frac{exp(2u') - 1}{exp(2u') + 1} \tag{6}$$

[6, p 406], where

$$l', u' = Z \pm z_{\frac{\alpha}{2}} \sqrt{\frac{1}{n-3}} \tag{7}$$

[6, p 406]. α denotes the desired alpha level of the confidence interval, whereas n specifies the size of the group the correlation is based on.

Tests for Comparison of Two Overlapping Correlations Based on Dependent Groups

The function `cocor.dep.groups.overlap()` implements tests for the comparison of two overlapping correlations based on dependent groups. In the following, r_{jk} and r_{jh} are the two correlations that are being compared; Z_{jk} and Z_{jh} are their Z transformed equivalents. r_{kh} is the related correlation that is additionally required. n specifies the size of the group the two correlations are based on.

pearson1898: Pearson and Filon's [7] z

This test was proposed by Pearson and Filon [7, p 259, formula xxxvii]. The test statistic z is computed as

$$z = \frac{\sqrt{n}(r_{jk} - r_{jh})}{\sqrt{(1 - r_{jk}^2)^2 + (1 - r_{jh}^2)^2 - 2k}} \tag{8}$$

[8, p 246, formula 4], where

$$k = r_{kh}(1 - r_{jk}^2 - r_{jh}^2) - \frac{1}{2}(r_{jk} r_{jh})(1 - r_{jk}^2 - r_{jh}^2 - r_{kh}^2) \tag{9}$$

[8, p 245, formula 3].

hotelling1940: Hotelling's [9] t

The test statistic t is given by

$$t = \frac{(r_{jk} - r_{jh})\sqrt{(n-3)(1+r_{kh})}}{\sqrt{2|R|}} \tag{10}$$

[9, p 278, formula 7] with $df = n - 3$, where

$$|R| = 1 + 2r_{jk}r_{jh}r_{kh} - r_{jk}^2 - r_{jh}^2 - r_{kh}^2 \tag{11}$$

[9, p 278]. Equation 10 is also given in Steiger [8, p 246], Glass and Stanley [10, p 311, formula 15.7], and Hittner et al. [11, p 152].

williams1959: Williams' [12] t

This test is a modification of Hotelling's [9] t and was suggested by Williams [12]. Two mathematically different formulae for Williams' t can be found in the literature [11, p 152]. This is the version that Hittner et al. [11, p 152] labeled as "standard Williams' t":

$$t = (r_{jk} - r_{jh})\sqrt{\frac{(n-1)(1+r_{kh})}{2(\frac{n-1}{n-3})|R| + \bar{r}^2(1-r_{kh})^3}} \tag{12}$$

with $df = n - 3$, where

$$\bar{r} = \frac{r_{jk} + r_{jh}}{2} \tag{13}$$

and

$$|R| = 1 + 2r_{jk}r_{jh}r_{kh} - r_{jk}^2 - r_{jh}^2 - r_{kh}^2. \tag{14}$$

An alternative formula for Williams' t – termed as "Williams' modified t per Hendrickson, Stanley, and Hills" [13] by Hittner et al. [11, p 152] – is implemented in `cocor` as `hendrickson1970` (see Equation 18 below). Equation 12 is also given in Steiger [8, p 246, formula 7] and Neill and Dunn [14, p 533].

Results from Equation 12 are in accordance with the results of DEPCORR [15] and DEPCOR [16]. However, we found several typographical errors in formulae that also claim to compute Williams' t. For example, the formula reported by Boyer, Palachek, and Schucany [17, p 76] contains an error because

the term $(1 - r_{rk})$ is not being cubed. There are also typographical errors in the formula described by Hittner et al. [11, p 152]. For example, $r_{jk} - r_{jh}$ is divided instead of being multiplied by the square root term, and in the denominator of the fraction in the square root term, there are additional parentheses so that the whole denominator is multiplied by 2. These same errors can also be found in Wilcox and Tian [18, p 107, formula 1].

olkin1967: Olkin's [19] z

In the original article by Olkin [19, p 112] and in Hendrickson et al. [13, p 190, formula 2], the reported formula contains a typographical error. Hendrickson and Collins [20, p 639] provide a corrected version. In the revised version, however, n in the enumerator is decreased by 1. The `cocor` package implements the corrected formula without the decrement. The formula implemented in `cocor` is used by Glass and Stanley [21, p 313, formula 14.19], Hittner et al. [11, p 152], and May and Hittner [22, p 259] [23, p 480]:

$$z = \frac{(r_{jk} - r_{jh})\sqrt{n}}{\sqrt{(1 - r_{jk}^2)^2 + (1 - r_{jh}^2)^2 - 2r_{kh}^3 - (2r_{kh} - r_{jk}r_{jh})(1 - r_{kh}^2 - r_{jk}^2 - r_{jh}^2)}}. \tag{15}$$

dunn1969: Dunn and Clark's [24] z

The test statistic z of this test is calculated as

$$z = \frac{(Z_{jk} - Z_{jh})\sqrt{n-3}}{\sqrt{2-2c}} \tag{16}$$

[24, p 370, formula 15], where

$$c = \frac{r_{kh}(1 - r_{jk}^2 - r_{jh}^2) - \frac{1}{2}r_{jk}r_{jh}(1 - r_{jk}^2 - r_{jh}^2 - r_{kh}^2)}{(1 - r_{jk}^2)(1 - r_{jh}^2)} \tag{17}$$

[24, p 368, formula 8].

hendrickson1970: Hendrickson, Stanley, and Hills [13] modification of Williams' [12] t

This test is a modification of Hotelling's [9] t and was suggested by Williams [12]. Two mathematically different formulae of Williams' [12] t can be found in the literature. `hendrickson1970` is the version that Hittner et al. [11, p 152] labeled as "Williams' modified t per Hendrickson, Stanley, and Hills" [13].

An alternative formula termed as "standard Williams' t" by Hittner et al. [11, p 152] is implemented as `williams1959` (see Equation 12 above). The `hendrickson1970` formula can be found in Hendrickson et al. [13, p 193], May and Hittner [22, p 259] [23, p 480], and Hittner et al. [11, p 152]:

$$t = \frac{(r_{jk} - r_{jh})\sqrt{(n-3)(1+r_{kh})}}{\sqrt{2|R| + \frac{(r_{jk}-r_{jh})^2(1-r_{kh})^3}{4(n-1)}}}, \tag{18}$$

with $df = n - 3$. A slightly changed version of this formula was provided by Dunn and Clark [25, p 905, formula 1.2], but seems to be erroneous, due to an error in the denominator.

steiger1980: Steiger's [8] modification of Dunn and Clark's [24] z using average correlations

This test was proposed by Steiger [8] and is a modification of Dunn and Clark's [24] z. Instead of r_{jk} and r_{jh}, the mean of the two is used. The test statistic z is defined as

$$z = \frac{(Z_{jk} - Z_{jh})\sqrt{n-3}}{\sqrt{2-2c}} \tag{19}$$

[8, p 247, formula 14], where

$$\bar{r} = \frac{r_{jk} + r_{jh}}{2} \tag{20}$$

[8, p 247] and

$$c = \frac{r_{kh}(1-2\bar{r}^2) - \frac{1}{2}\bar{r}^2(1-2\bar{r}^2-r_{kh}^2)}{(1-\bar{r}^2)^2} \tag{21}$$

[8, p 247, formula 10; in the original article, there are brackets missing around the divisor].

meng1992: Meng, Rosenthal, and Rubin's [26] z

This test is based on the test statistic z,

$$z = (Z_{jk} - Z_{jh})\sqrt{\frac{n-3}{2(1-r_{kh})h}}, \tag{22}$$

[26, p 173, formula 1], where

$$h = \frac{1 - f\overline{r^2}}{1 - \overline{r^2}} \tag{23}$$

[26, p 173, formula 2],

$$f = \frac{1 - r_{kh}}{2(1 - \overline{r^2})} \tag{24}$$

(f must be ≤ 1) [26, p 173, formula 3], and

$$\overline{r^2} = \frac{r_{jk}^2 + r_{jh}^2}{2} \tag{25}$$

[26, p 173]. This test also constructs a confidence interval of the difference between the two correlation coefficients r_{jk} and r_{jh}:

$$L, U = Z_{jk} - Z_{jk} \pm z_{\frac{\alpha}{2}} \sqrt{\frac{2(1 - r_{kh})h}{n - 3}} \tag{26}$$

[26, p 173, formula 4]. α denotes the desired alpha level of the confidence interval. If the confidence interval includes zero, the null hypothesis that the two correlations are equal must be retained. If the confidence interval does not include zero, the null hypothesis has to be rejected.

hittner2003: Hittner, May, and Silver's [11] modification of Dunn and Clark's [24] z using a backtransformed average Fisher's [2] Z procedure

The approach to backtransform averaged Fisher's [2] Zs was first proposed by Silver and Dunlap [27] and was applied to the comparison of overlapping correlations by Hittner et al. [11]. The test is based on Steiger's [8] approach. The test statistic z is calculated as

$$z = \frac{(Z_{jk} - Z_{jh})\sqrt{n - 3}}{\sqrt{2 - 2c}} \tag{27}$$

[11, p 153], where

$$c = \frac{r_{kh}(1 - 2\bar{r}_z^2) - \frac{1}{2}\bar{r}_z^2(1 - 2\bar{r}_z^2 - r_{kh}^2)}{(1 - \bar{r}_z^2)^2} \tag{28}$$

[11, p 153],

$$\bar{r}_z = \frac{exp(2\bar{Z} - 1)}{exp(2\bar{Z} + 1)} \tag{29}$$

[27, p 146, formula 4], and

$$\bar{Z} = \frac{Z_{jk} + Z_{jh}}{2} \tag{30}$$

[27, p 146].

zou2007: Zou's [6] confidence interval

This test calculates the confidence interval of the difference between the two correlation coefficients r_{jk} and r_{jh}. If the confidence interval includes zero, the null hypothesis that the two correlations are equal must be retained. If zero is outside the confidence interval, the null hypothesis has to be rejected. A lower and upper bound for the interval (L and U, respectively) is given by

$$L = r_{jk} - r_{jh} - \sqrt{(r_{jk} - l_1)^2 + (u_2 - r_{jh})^2 - 2c(r_{jk} - l_1)(u_2 - r_{jh})} \quad (31)$$

and

$$U = r_{jk} - r_{jh} + \sqrt{(u_1 - r_{jk})^2 + (r_{jh} - l_2)^2 - 2c(u_1 - r_{jk})(r_{jh} - l_2)} \quad (32)$$

[6, p 409], where

$$l = \frac{exp(2l') - 1}{exp(2l') + 1}, \quad (33)$$

$$u = \frac{exp(2u') - 1}{exp(2u') + 1} \quad (34)$$

[6, p 406],

$$c = \frac{(r_{kh} - \frac{1}{2}r_{jk}r_{jh})(1 - r_{jk}^2 - r_{jh}^2 - r_{kh}^2) + r_{kh}^3}{(1 - r_{jk}^2)(1 - r_{jh}^2)} \quad (35)$$

[6, p 409], and

$$l', u' = Z \pm z_{\frac{\alpha}{2}} \sqrt{\frac{1}{n-3}} \quad (36)$$

[6, p 406]. α denotes the desired alpha level of the confidence interval.

Tests for Comparison of Two Nonoverlapping Correlations Based on Dependent Groups

The function `cocor.dep.groups.nonoverlap()` implements tests for the comparison of two nonoverlapping correlations based on dependent groups. In the following, r_{jk} and r_{hm} are the two correlations that are being compared; Z_{jk} and Z_{hm} are their Z transformed equivalents. r_{jh}, r_{kh}, r_{jm}, and r_{km} are the related correlations that are also required. n specifies the size of the group the two correlations are based on.

pearson1898: Pearson and Filon's [7] z

This test was proposed by Pearson and Filon [7, p 262, formula xl]. The formula for the test statistic z is computed as

$$z = \frac{\sqrt{n}(r_{jk} - r_{hm})}{\sqrt{(1 - r_{jk}^2)^2 + (1 - r_{hm}^2)^2 - k}} \tag{37}$$

[28, p 179, formula 1], where

$$\begin{aligned} k = &(r_{jh} - r_{jk}r_{kh})(r_{km} - r_{kh}r_{hm}) + (r_{jm} - r_{jh}r_{hm})(r_{kh} - r_{jk}r_{jh}) \\ &+(r_{jh} - r_{jm}r_{hm})(r_{km} - r_{jk}r_{jm}) + (r_{jm} - r_{jk}r_{km})(r_{kh} - r_{km}r_{hm}) \end{aligned} \tag{38}$$

[28, p 179, formula 2]. The two formulae can also be found in Steiger [8, p 245, formula 2 and p. 246, formula 5].

dunn1969: Dunn and Clark's [24] z

The test statistic z of this test is calculated as

$$z = \frac{(Z_{jk} - Z_{hm})\sqrt{n-3}}{\sqrt{2 - 2c}} \tag{39}$$

[24, p 370, formula 15], where

$$\begin{aligned} c = \Big(&\frac{1}{2}r_{jk}r_{hm}(r_{jh}^2 + r_{jm}^2 + r_{kh}^2 + r_{km}^2) + r_{jh}r_{km} + r_{jm}r_{kh} \\ &-(r_{jk}r_{jh}r_{jm} + r_{jk}r_{kh}r_{km} + r_{jh}r_{kh}r_{hm} + r_{jm}r_{km}r_{hm})\Big) \\ &\Big/\Big((1 - r_{jk}^2)(1 - r_{hm}^2)\Big) \end{aligned} \tag{40}$$

[24, p 368, formula 9].

steiger1980: Steiger's [8] modification of Dunn and Clark's [24] z using average correlations

This test was proposed by Steiger [8] and is a modification of Dunn and Clark's [24] z. Instead of r_{jk} and r_{hm} the mean of the two is being used. The test statistic z is given by

$$z = \frac{(Z_{jk} - Z_{hm})\sqrt{n-3}}{\sqrt{2-2c}} \tag{41}$$

[8, p 247, formula 15], where

$$\bar{r} = \frac{r_{jk} + r_{hm}}{2} \tag{42}$$

[8, p 247] and

$$\begin{aligned} c = \Big(&\frac{1}{2}\bar{r}^2(r_{jh}^2 + r_{jm}^2 + r_{kh}^2 + r_{km}^2) + r_{jh}r_{km} + r_{jm}r_{kh} \\ &-(\bar{r}r_{jh}r_{jm} + \bar{r}r_{kh}r_{km} + r_{jh}r_{kh}\bar{r} + r_{jm}r_{km}\bar{r})\Big) \\ &\Big/(1-\bar{r}^2)^2 \end{aligned} \tag{43}$$

[8, p 247, formula 11; in the original article, there are brackets missing around the divisor].

raghunathan1996: Raghunathan, Rosenthal, and Rubin's [28] modification of Pearson and Filon's [7] z

This test of Raghunathan et al. [28] is based on Pearson and Filon's [7] z. Unlike Pearson and Filon [7], Raghunathan et al. [28] use Z transformed correlation coefficients. The test statistic z is computed as

$$z = \sqrt{\frac{n-3}{2}} \frac{Z_{jk} - Z_{hm}}{\sqrt{1 - \frac{k}{2(1-r_{jk}^2)(1-r_{hm}^2)}}} \tag{44}$$

[28, p 179, formula 3], where

$$\begin{aligned} k = &(r_{jh} - r_{jk}r_{kh})(r_{km} - r_{kh}r_{hm}) + (r_{jm} - r_{jh}r_{hm})(r_{kh} - r_{jk}r_{jh}) \\ &+(r_{jh} - r_{jm}r_{hm})(r_{km} - r_{jk}r_{jm}) + (r_{jm} - r_{jk}r_{km})(r_{kh} - r_{km}r_{hm}) \end{aligned} \tag{45}$$

[28, p 179, formula 2].

silver2004: Silver, Hittner, and May's [29] modification of Dunn and Clark's [24] z using a backtransformed average Fisher's [2] Z procedure

The approach to backtransform averaged Fisher's [2] Zs was first proposed in Silver and Dunlap [27] and was applied to the comparison of nonoverlapping correlations by Silver et al. [29]. The test is based on Steiger's [8] approach. The formula of the test statistic z is given by

$$z = \frac{(Z_{jk} - Z_{hm})\sqrt{n-3}}{\sqrt{2-2c}} \tag{46}$$

[29, p 55, formula 5], where

$$\begin{aligned} c = \Big(&\frac{1}{2}\bar{r}_z^2(r_{jh}^2 + r_{jm}^2 + r_{kh}^2 + r_{km}^2) + r_{jh}r_{km} + r_{jm}r_{kh} \\ &-(\bar{r}_z r_{jh} r_{jm} + \bar{r}_z r_{kh} r_{km} + r_{jh} r_{kh} \bar{r}_z + r_{jm} r_{km} \bar{r}_z)\Big) \\ &\Big/(1-\bar{r}_z^2)^2 \end{aligned} \tag{47}$$

[29, p 56],

$$\bar{r}_z = \frac{exp(2\bar{Z}-1)}{exp(2\bar{Z}+1)} \tag{48}$$

[27, p 146, formula 4], and

$$\bar{Z} = \frac{Z_{jk} + Z_{hm}}{2} \tag{49}$$

[29, p 55].

zou2007: Zou's [6] confidence interval

This test calculates the confidence interval of the difference between the two correlations r_{jk} and r_{hm}. If the confidence interval includes zero, the null hypothesis that the two correlations are equal must be retained. If the confidence interval does not include zero, the null hypothesis has to be rejected. A lower and upper bound for the interval (L and U, respectively) is given by

$$L = r_{jk} - r_{hm} - \sqrt{(r_{jk} - l_1)^2 + (u_2 - r_{hm})^2 - 2c(r_{jk} - l_1)(u_2 - r_{hm})} \tag{50}$$

and

$$U = r_{jk} - r_{hm} + \sqrt{(u_1 - r_{jk})^2 + (r_{hm} - l_2)^2 - 2c(u_1 - r_{jk})(r_{hm} - l_2)} \tag{51}$$

[6, pp 409–410], where

$$l = \frac{exp(2l') - 1}{exp(2l') + 1}, \tag{52}$$

$$u = \frac{exp(2u') - 1}{exp(2u') + 1} \tag{53}$$

[6, p 406],

$$\begin{aligned} c = \Big(&\frac{1}{2} r_{jk} r_{hm} (r_{jh}^2 + r_{jm}^2 + r_{kh}^2 + r_{km}^2) + r_{jh} r_{km} + r_{jm} r_{kh} \\ &- (r_{jk} r_{jh} r_{jm} + r_{jk} r_{kh} r_{km} + r_{jh} r_{kh} r_{hm} + r_{jm} r_{km} r_{hm})\Big) \\ &\Big/ \Big((1 - r_{jk}^2)(1 - r_{hm}^2)\Big) \end{aligned} \tag{54}$$

[6, p 409], and

$$l', u' = Z \pm z_{\frac{\alpha}{2}} \sqrt{\frac{1}{n-3}} \tag{55}$$

[6, p 406]. α denotes the desired alpha level of the confidence interval.

References

1. R Core Team. R: A Language and Environment for Statistical Computing. Vienna, Austria; 2014. Available: http://www.R-project.org. Accessed 21 February 2015.
2. Fisher RA. On the Probable Error of a Coefficient of Correlation Deduced From a Small Sample. Metron. 1921;1: 3–32. Available: http://hdl.handle.net/2440/15169. Accessed 21 February 2015.
3. Fisher RA. Statistical Methods for Research Workers. Edinburgh, Scotland: Oliver and Boyd; 1925. Available: http://psychclassics.yorku.ca. Accessed 21 February 2015.
4. Peters CC, van Voorhis WR. Statistical Procedures and Their Mathematical Bases. New York, NJ: McGraw-Hill; 1940.
5. Cohen J, Cohen P, West SG, Aiken LS. Applied Multiple Regression/Correlation Analysis for the Behavioral Sciences. 3rd ed. Mahwah, NJ: Erlbaum; 2003.
6. Zou GY. Toward Using Confidence Intervals to Compare Correlations. Psychol Methods. 2007;12: 399–413. doi: 10.1037/1082-989X.12.4.399
7. Pearson K, Filon LNG. Mathematical Contributions to Theory of Evolution: IV. On the Probable Errors of Frequency Constants and on the Influence of Random Selection and Correlation. Philos Trans R Soc Lond A. 1898;191: 229–311. doi: 10.1098/rsta.1898.0007
8. Steiger JH. Tests for Comparing Elements of a Correlation Matrix. Psychol Bull. 1980;87: 245–251. doi: 10.1037//0033-2909.87.2.245
9. Hotelling H. The Selection of Variates for Use in Prediction, with Some Comments on the General Problem of Nuisance Parameters. Ann Math Stat. 1940;11: 271–283. doi: 10.1214/aoms/1177731867
10. Glass GV, Stanley JC. Statistical Methods in Education and Psychology. 2nd ed. Englewood Cliffs, NJ: Prentice-Hall; 1984.
11. Hittner JB, May K, Silver NC. A Monte Carlo Evaluation of Tests for Comparing Dependent Correlations. J Gen Psychol. 2003;130: 149–168. doi: 10.1080/00221300309601282

12. Williams EJ. The Comparison of Regression Variables. J R Stat Soc B. 1959;21: 396–399. Available: `http://www.jstor.org/stable/2983809`. Accessed 21 February 2015.

13. Hendrickson GF, Stanley JC, Hills JR. Olkin's New Formula for Significance of r13 vs. r23 Compared with Hotelling's Method. Am Educ Res J. 1970;7: 189–195. doi: 10.2307/1162159

14. Neill JJ, Dunn OJ. Equality of Dependent Correlation Coefficients. Biometrics. 1975;31: 531–543. doi: 10.2307/2529435

15. Hittner JB, May K. DEPCORR: A SAS Program for Comparing Dependent Correlations. Appl Psychol Meas. 1998;22: 93–94. doi: 10.1177/01466216980221010

16. Silver NC, Hittner JB, May K. A FORTRAN 77 Program for Comparing Dependent Correlations. Appl Psychol Meas. 2006;30: 152–153. doi: 10.1177/0146621605277132

17. Boyer IE, Palachek AD, Schucany WR. An Empirical Study of Related Correlation Coefficients. J Educ Stat. 1983;8: 75–86. doi: 10.2307/1164871

18. Wilcox RR, Tian T. Comparing Dependent Correlations. J Gen Psychol. 2008;135: 105–112. doi: 10.3200/GENP.135.1.105-112

19. Olkin I. Correlations Revisited. In: Stanley JC, editor. Improving Experimental Design and Statistical Analysis. Chicago, IL: Rand McNally; 1967. pp. 102–128.

20. Hendrickson GF, Collins JR. Note Correcting the Results in 'Olkin's New Formula for the Significance of r13 vs. r23 Compared with Hotelling's Method'. Am Educ Res J. 1970;7: 639–641. doi: 10.2307/1161847

21. Glass GV, Stanley JC. Statistical Methods in Education and Psychology. Englewood Cliffs, NJ: Prentice-Hall; 1970.

22. May K, Hittner JB. A Note on Statistics for Comparing Dependent Correlations. Psychol Rep. 1997;80: 475–480. doi: 10.2466/pr0.1997.80.2.475

23. May K, Hittner JB. Tests for Comparing Dependent Correlations Revisited: A Monte Carlo Study. J Exp Educ. 1997;65: 257–269. doi: 10.1080/00220973.1997.9943458

24. Dunn OJ, Clark VA. Correlation Coefficients Measured on the Same Individuals. J Am Stat Assoc. 1969;64: 366–377. doi: 10.2307/2283746

25. Dunn OJ, Clark VA. Comparison of Tests of the Equality of Dependent Correlation Coefficients. J Am Stat Assoc. 1971;66: 904–908. doi: 10.2307/2284252

26. Meng XL, Rosenthal R, Rubin DB. Comparing Correlated Correlation Coefficients. Psychol Bull. 1992;111: 172–175. doi: 10.1037//0033-2909.111.1.172

27. Silver NC, Dunlap WP. Averaging Correlation Coefficients: Should Fisher's Z Transformation Be Used? J Appl Psychol. 1987;72: 146–148. doi: 10.1037//0021-9010.72.1.146

28. Raghunathan TE, Rosenthal R, Rubin DB. Comparing Correlated but Nonoverlapping Correlations. Psychol Methods. 1996;1: 178–183. doi: 10.1037//1082-989X.1.2.178

29. Silver NC, Hittner JB, May K. Testing Dependent Correlations with Nonoverlapping Variables: A Monte Carlo Simulation. J Exp Educ. 2004;73: 53–69. doi: 10.3200/JEXE.71.1.53-70
